# Design of a Smartphone Indoor Positioning Dynamic Ground Truth Reference System Using Robust Visual Encoded Targets

**DOI:** 10.3390/s19051261

**Published:** 2019-03-12

**Authors:** Xuan Liao, Ruizhi Chen, Ming Li, Bingxuan Guo, Xiaoji Niu, Weilong Zhang

**Affiliations:** 1 State Key Laboratory of Information Engineering in Surveying Mapping and Remote Sensing, Wuhan University, Wuhan 430079, China; liaoxuan@whu.edu.cn (X.L.); 00201550@whu.edu.cn (B.G.); zhangweilong@whu.edu.cn (W.Z.); 2 School of Resource and Environmental Science, Wuhan University, Wuhan 430079, China; 3 GNSS Research Center, Wuhan University, Wuhan 430079, China; xjniu@whu.edu.cn

**Keywords:** smartphone, indoor positioning, visual encoded target, ground truth reference system

## Abstract

Smartphone indoor positioning ground truth is difficult to directly, dynamically, and precisely measure in real-time. To solve this problem, this paper proposes and implements a robust smartphone high-precision indoor positioning dynamic real-time ground truth reference system using color visual scatter-encoded targets based on machine vision and photogrammetry. First, a kind of novel high-precision color vision scatter-encoded patterns with a robust recognition rate is designed. Then we use a smartphone to obtain a sequence of images of an experimental room and extract the base points of the color visual scatter-encoded patterns from the sequence images to establish the indoor local coordinate system of the encoded targets. Finally, we use a high-efficiency algorithm to decode the targets of a real-time dynamic shooting image to obtain accurate instantaneous pose information of a smartphone camera and establish the high-precision and high-availability smartphone indoor positioning direct ground truth reference system for preliminary real-time accuracy evaluation of other smartphone positioning technologies. The experimental results show that the encoded targets of the color visual scatter-encoded pattern designed in this paper are easy to detect and identify, and the layout is simple and affordable. It can accurately and quickly solve the dynamic instantaneous pose of a smartphone camera to complete the self-positioning of the smartphone according to the artificial scatter feature visual positioning technology. It is a fast, efficient and low-cost accuracy-evaluation method for smartphone indoor positioning.

## 1. Introduction

A survey show that people spend more than 70% of their life indoors [1]. Indoor spaces that people frequently enter and exit, such as airports, stations, supermarkets, hospitals, shopping centers, museums, libraries, and underground parking lots, have a high demand for location services. Based on smartphone outdoor positioning technology, positioning accuracy is less than 1 m with the help of a Global Navigation Satellite System (GNSS) signal. However, high-availability indoor positioning accuracy is between two and three meters, based on current technologies. Scientists and industrial researchers in many countries are working on indoor positioning technology with a highly available accuracy of 1 m [2]. Indoor positioning methods for smartphones are mainly based on active radio frequency signals such as Wi-Fi and Bluetooth, and built-in sensors for indoor positioning such as geomagnetism, inertial navigation, and vision [3]. The indoor positioning ground truth reference system is an important basis for evaluating the positioning accuracy of smartphone indoor positioning technologies. The system used in this work is based on three kinds of measurement methods: high-precision measurement robot (automatic total station), high-precision laser Simultaneous Localization and Mapping (SLAM), and high-precision inertial navigation, which can provide the precise position and orientation of a smartphone. A measuring robot is generally used to statically measure the position of a smartphone, and its measuring value is used as the ground truth to evaluate indoor positioning accuracy [4]. However, a high-precision Inertial Measurement Unit (IMU) must be tied to the smartphone to measure its real-time pose. The volume and weight of a high-precision IMU, along with its additional power supply, make it difficult to implement in actual measurement. A post-evaluation precision system based on an expensive high-precision laser SLAM utilizes 3D SLAM technology, such as NavVis M3/6, to provide high-precision indoor motion trajectories as the positioning ground truth to compare with smartphone positioning results in the same trajectories. However, it is an offsite evaluation system with a high price, and data processing is quite complicated [5]. There are also three-dimensional target motion analysis systems based on an infrared high-speed camera, and other methods, such as the U.S. OptiTrack and Swedish Qualisys motion-capture systems, which are generally used to capture human body motion to obtain centimeter-level accuracy poses of a measured target. However, these systems require professionals to establish an experimental platform and environment, and there are other problems, such as expensive equipment, a complex layout, and small application scenarios. These systems are not suitable to frequently measure smartphone poses in a large scene in different environments, and the cost-effectiveness ratio is quite low [6,7].

The problems of directly and dynamically measuring a smartphone pose using current indoor positioning ground truth reference systems include high prices layout and cumbersome and complex operation. To address these problems, we propose a high-precision ground truth reference system of indoor visual positioning, based on color visual scatter-encoded patterns. This system combines with the meter-level experimental accuracy of current smartphone indoor positioning methods. The method decodes color visual scatter-encoded patterns of images through a single image captured by a smartphone, and directly obtains its pose ground truth to realize self-positioning. It solves the problems that it is hard to directly dynamically measure a smartphone pose, and its indoor positioning measurement value must be frequently compared to the ground truth reference system during evaluation of positioning accuracy. It simplifies the workflow of the initial accuracy evaluation of positioning technology, and it has a low cost and relatively high precision.

## 2. Related Work

The encoded target pattern is an artificial target with its own specific information. It is unique and recognizable, and has been widely used in the machine vision, photogrammetry, augmented reality, and other fields [8,9,10,11,12]. Commonly used artificial target types include concentric ring-based encoded targets, dot-based distribution encoded targets, or color-based encoded targets [13,14,15,16]. Concentric ring-based encoded targets are usually based on binary encoding, and mainly have two parts: positioning targets at the center and a concentric ring encoded band [17]. This target has the advantages of simple principles and easy recognition, but it is not suitable for a large number of encoding bits because of the size limitation, so its encoding capacity is usually small. The latter issue has been addressed by adding one or more bands, which constitute a dual-loop (multi-ring) target to extend the encoding capacity [18,19]. The encoded target must extract both the central circular targets and the ring encoded targets during recognition, so its decoding process is relatively complex. A dot-distributed gray-scale encoded target, based mainly on the distribution relationship of circular targets, has been proposed [20]. However, it still needs a certain amount of calculation when distinguishing dots of different attributes, and it can incorrectly recognize a target when the camera has a large tilt angle or distortion. A color encoded targets was designed to measure high-precision three-dimensional objects [21]. Compared with gray-encoded targets, color encoded targets improve the recognition rate by making. Nevertheless, their structure is more complicated, and more geometric figures must be extracted. Furthermore, the number of complete encoded sets is relatively limited. The traditional gray-encoded target is generally identified by its relationship of geometry and structure, and it is prone to incorrect recognition due to the large inclination and distortion of images. A color encoded target adds color information, but its structure is relatively complicated, which increases the complexity of the algorithm with the problem of a low recognition rate. This paper addresses the above problems by combining the color and geometric information in encoded targets, by designing a set of color visual scatter encoded patterns for fast calculation and high reliability. Hence, the positioning accuracy of a single smartphone image can reach the centimeter level, which is far superior to other indoor other positioning methods, such as Bluetooth, WiFi, 4G, and Pedestrian Dead Reckoning (PDR), enabling encoded targets to meet accuracy requirements of the ground truth reference system.

## 3. Design of Visual Scatter-Encoded Targets

Encoded targets have different shapes, such as circles, squares, and triangles [22,23,24]. An experiment showed that the edge of a circular target is the most rounded, and there is no change of rotation angle when rotating in the plane [25]. Furthermore, in the orthographic condition, the distances between the center and the edge points are equal. This target is easily identified and located, and it is used in logistics warehousing and precision control of indoor robots. This paper uses a circular pattern shape for encoded targets.

**Figure 1:**
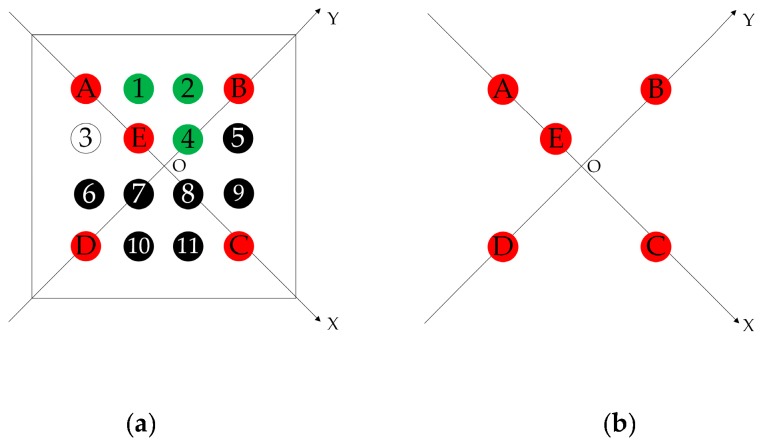
The encoding principle. (**a**) Structure of color-encoded target pattern. (**b**) Base points coordinates.

As shown in Figure 1a, the encoded target patterns designed in this paper arrange the color circular scatter points in a 4 × 4 configuration with a consistent shape and size. Color scatter-encoded targets can be divided into two categories: base points and identification points. An encoded target pattern has five base points, consisting of color scatter points A, B, C, and D at the corners, plus a near corner point E in Figure 1a, while the identification points are located in the remaining position. Figure 1b shows the coordinates formed by the base points. The position, shape, and color of point A, B, C, D and E are unchanged, and the structure retains the unchanged affine transformation parameters after affine transformation so that it has invariance of scaling, rotation and translation. During acquisition, recognition, and decoding of indoor visual encoded targets, the RGB value of the captured indoor image may be untrue due to the influence of an external light source, error of the camera’s photosensitive chip, and other factors. To improve the accuracy of color recognition and reduce the interference of the above factors, it is necessary to enhance the contrast between different colors. The three primary colors and their complementary colors has been shown to improve the color recognition rate [26]. We choose red, white, black, and green to improve the accuracy of color recognition by combining the geometric structure characteristics of the designed encoded targets. In addition, a white background color will result in strong reflection of images in this encoding method, which affects the photosensitive effect of encoded targets. However, the image reflection is weak with a black background, and encoded targets will be less affected by reflection when imaging.

**Figure 2:**
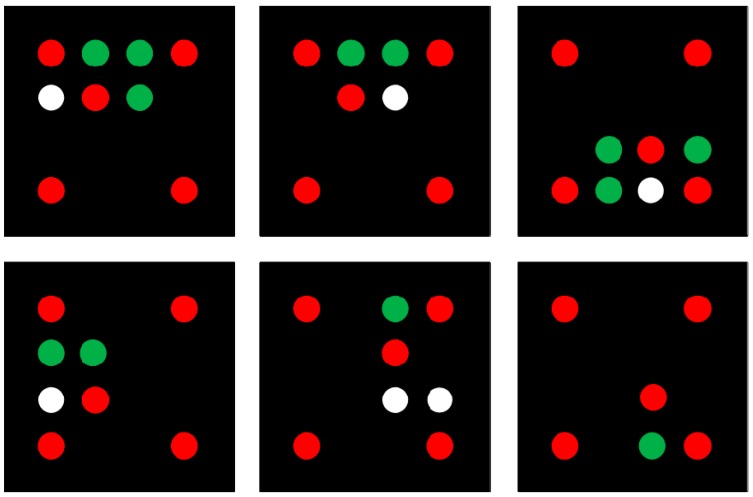
Examples of color scatter-encoded target patterns.

Therefore, the edges of non-black circular encoded targets are clearer with a black background than with a white background. This is the basis of the encoded target patterns designed in this paper with a black background. Figure 2 shows some examples of the color scatter-encoded target pattern designed in this paper. Base points are red circles, and identification points are white, green, and black circles. Combining with geometric structure and color information, the number of color scatter encoded target patterns can reach 177,147, which can meet the application requirements of most scenarios. Moreover, our method has the invariance of scaling, rotation and translation, and a stable, high recognition rate and sufficient encoding capacity.

## 4. Dynamic Truth Reference System Based on Visual Encoded Targets

### 4.1. Extraction of Base Points

Based on a complete sequence of smartphone images of an indoor experimental scene, we extract base points from the acquired images. Before extraction, we deepen the RGB value of red base points through a custom channel. Since the Canny operator can produce single-pixel edges and is less sensitive to noise, the Canny operator is suitable to extract the edges of circular artificial target patterns. Therefore, we use Canny edge detection [27] to detect the edges of acquired images to obtain a binarized image. Since a circular target is generally imaged as an ellipse due to projection transformation, we use the ellipse fitting to locate the image center of base points. The position information of the edge pixel is used to determine the center of the base point by fitting an elliptic equation, and then the elliptic sequence is established. The general equation for an ellipse in a plane is on below:

(1) $$x^{2}+2Bxy+Cy^{2}+2Dx+2Ey+F=0.$$

In Equation (1), (*x*, *y*) are the central coordinates of the ellipse, and *B*, *C*, *D*, *E*, and *F* are five parameters of the elliptic equation. These parameters are obtained by ellipse fitting, and the elliptic central coordinates (*x*_0_, *y*_0_) can be obtained from Equation (2):

(2) $$\{\begin{array}{c} x_{0}=\frac{BE-CD}{C-B^{2}},\\ y_{0}=\frac{BD-E}{C-B^{2}}. \end{array}$$

To avoid false base points, after recognition complete, we must judge whether it conforms to circular or elliptical pattern characteristics. This takes place in two steps. First, we check whether the ratio of the semi-major and semi-minor axes are within a certain range. Second, the elliptical area cannot be too small. Experiments show that the elliptical area should be greater than 10 pixels value to eliminate false base points.

### 4.2. Setting Up a Local Coordinate System

After extracting base points of the sequence images, the local coordinate system of the color scatter-encoded target pattern is established according to its base point coordinates. A base point ellipse is randomly selected, and the Euler distance *S_i_* between it and other base point ellipses is calculated, centered on its central coordinate (*x_0_*, *y_0_*), as follows:

(3) $$S_{i}=\sqrt{{\left(x_{i}-x_{0}\right)}^{2}+{\left(y_{i}-y_{0}\right)}^{2}},$$

where *x_i_* and *y_i_* are respectively the values in the *x* and *y* directions of other elliptical center coordinates.

A temporary set of candidate ellipses to be detected is composed of this base point ellipse and four nearby base point ellipses. When these five base point ellipses meet the following principles, they are considered to possibly belong to the same color scatter-encoded target pattern, and otherwise they should be removed:

(1) No two ellipses can have an inclusive or intersecting relationship;
(2) The maximum semi-major axis radius of the ellipses cannot be greater than twice the minimum semi-minor axis radius.

**Figure 3:**
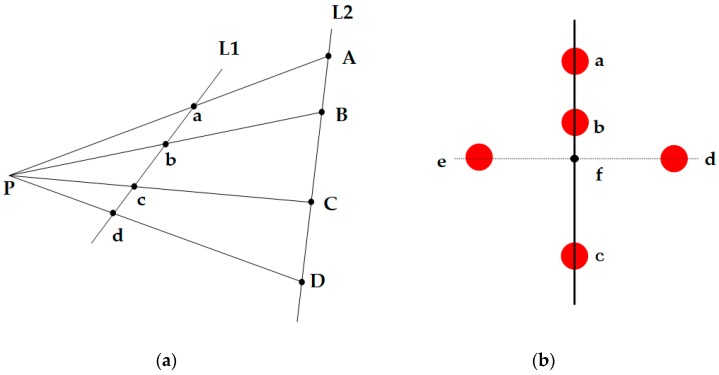
Structure principle of base points. (**a**) Invariant of cross-ratio. (**b**) Local coordinate system of color encoded target pattern.

Among the five ellipses, we find the two that are farthest apart, and judge whether there are collinear ellipses among the remaining three. If so, this line is assumed to be *L_i_*, and we detect that whether there is an ellipse between the other ellipses. If not, then we must return to the previous step. The two ellipses of the longest distance between those need to be judged that whether those on the two sides of *L_i_*. If so, the ellipse is numbered to *a* in the Figure 3b, which is close to the one of two ellipses of the longest distance, and other ellipses are numbered to *b*, *c*, ***e*** and *d* in the Figure 3b. If not, then we return to the previous step. Then, we must judge whether the center point *f* of ellipses *d* and *e* are collinear with ellipses *a* and *c*. If so, the cross-ratio judgment is made on the center of ellipses *a*, *b*, *c* and the *f* point. Figure 3a shows the principle of cross-ratio invariance. If four points *a*, *b*, *c*, and *d* are collinear with line L1, then they are projected onto line L2 through the projection center P, with corresponding image points A, B, C, and D. Their relationship is:

(4) (a,b;c,d)=(A,B;C,D),

where the cross-ratio of the four points is:

(5) $$\left(a,b;c,d\right)=\frac{ac/ad}{bc/bd}.$$

From the above two equations, it can be seen that the cross-ratio of the four points of the collinear line equals the cross-ratio of the corresponding image points, which indicates the invariance of the cross-ratio projection. As shown in Figure 3b, the cross-ratio *(ab*, *fc)* of the elliptical center of *a*, *b*, *c*, and point *f* is defined as:

(6) $$\left(ab,fc\right)=\frac{af\cdot bc}{ac\cdot bf}.$$

In Equation (6), *af*, *bc*, *ac*, and *bf* are all directed line segments, which are not distances. Based on Equation (3), the cross-ratio of this paper is set to 0.5; otherwise, we return to the previous step. If the cross-ratio is consistent with the threshold within the error range, then the five ellipses are the base points of the same color encoded target pattern, and the local coordinate system of the color encoded target pattern can be established. Otherwise, ellipses *a* and *c* must be selected again.

### 4.3. Building the Dataset of Encoded Targets

#### 4.3.1. Solution Object Coordinates

This paper utilizes the Photoscan software to process indoor images to obtain the intrinsics and extrinsics of a camera. The coordinates of object points corresponding to image points are obtained using the method of forward intersection. According to the coordinate values of four control points in the indoor local coordinate system, the coordinates of object points are transformed to the indoor local coordinate system.

#### 4.3.2. Decoding Encoded Targets

The maximum value Max*_i_* and minimum value Min*_i_* of the RGB channel of the five base points in the color encoded target pattern are counted. According to Max*_i_* and Min*_i_*, the pixels of base points of the same color encoded target pattern are linearly stretched, and their color is judged. Judging rules are as follows: If the three channel values are all less than 50, the target is considered to be black encoded target. If the largest value of the green channel is greater than 100, which is 50 higher than the value of the other two channels, it is considered to be the green encoded target. If the three channel values are all greater than 150 and the difference between each pairs of channels is not more than 50, the target is considered to be white encoded target. After attaching the color information to each encoded target, the color visual encoded target pattern is encoded. The state values of white, green, and black encoded targets are set to 2, 1, 0, respectively, and consist of a ternary code which is converted to decimal code to complete the decoding process. Finally, the decimal-encoded values of the color encoded target patterns and corresponding coordinate values are recorded in the dataset of encoded targets.

### 4.4. Solution Pose of Single Positioning Image

After completing the above work, the elements of exterior orientation of the positioning image can be calculated by space resection. Taking the photo plane and local indoor coordinate values of the color encoded target patterns as the observation and known value, respectively, the pose of the single positioning image is solved iteratively using the collinear equation, the least square method, and indirect adjustment. The calculation procedure is as follows: First, a single positioning image of a smartphone is decoded to obtain an encoded value of the color encoded target patterns, and the indoor coordinate value (*X_i_*, *Y_i_*) and photo coordinate value (*x_i_*, *y_i_*) are obtained from the color encoded target pattern dataset according to the encoded value of the color encoded target patterns. Then the collinear equation is expanded by Taylor series to obtain the linearized collinear equation. The collinear and the linearized collinear equation are shown in equations (7) and (8):

(7) $$\{\begin{array}{c} x-x_{0}=-f\frac{a_{1}\left(X_{A}-X_{S}\right)+b_{1}\left(Y_{A}-Y_{S}\right)+c_{1}\left(Z_{A}-Z_{S}\right)}{a_{3}\left(X_{A}-X_{S}\right)+b_{3}\left(Y_{A}-Y_{S}\right)+c_{3}\left(Z_{A}-Z_{S}\right)},\\ y-y_{0}=-f\frac{a_{2}\left(X_{A}-X_{S}\right)+b_{2}\left(Y_{A}-Y_{S}\right)+c_{2}\left(Z_{A}-Z_{S}\right)}{a_{3}\left(X_{A}-X_{S}\right)+b_{3}\left(Y_{A}-Y_{S}\right)+c_{3}\left(Z_{A}-Z_{S}\right)}. \end{array}$$

In Equation (7), *x*, *y* are image plane coordinate values of the image point. *f*, *x_0_,* and *y_0_* are the intrinsic parameters of the camera. *X_A_*, *Y_A_*, and *Z_A_* and *X_S_*, *Y_S_* and *Z_S_* are object coordinate values of the object point and camera, respectively. a_i_, b_i_, and c_i_ (i = 1, 2, 3) are nine directional cosines consisting of three angle elements of the extrinsic parameters:

(8) $$\{\begin{array}{c} x=\left(x\right)+\frac{\partial x}{\partial X_{s}}dX_{s}+\frac{\partial x}{\partial Y_{s}}dY_{s}+\frac{\partial x}{\partial Z_{s}}dZ_{s}+\frac{\partial x}{\partial \varphi }d\varphi +\frac{\partial x}{\partial \omega }d\omega +\frac{\partial x}{\partial \kappa }d\kappa ,\\ y=\left(y\right)+\frac{\partial y}{\partial X_{s}}dX_{s}+\frac{\partial y}{\partial Y_{s}}dY_{s}+\frac{\partial y}{\partial Z_{s}}dZ_{s}+\frac{\partial y}{\partial \varphi }d\varphi +\frac{\partial y}{\partial \omega }d\omega +\frac{\partial y}{\partial \kappa }d\kappa . \end{array}$$

In Equation (8), (*x*) and (*y*) are function approximations obtained by bringing the initial values *Xs_0_*, *Ys_0_*, Z*s_0_*, *φ_0_*, *ω_0_*, an *κ_0_* of the extrinsics into the collinear equation. *d_Xs_*, *d_Ys_*, *d_Zs_*, *d_φ_*, *d_ω_*, and *d_κ_* are correction values of the extrinsics approximation. *∂_x_*/*∂_Xs_ ∂_y_*/∂_κ_ are partial derivatives, which are the coefficients of the correction value of the extrinsics approximation. Then the error equation is:

(9) $$\{\begin{array}{c} v_{x}=\frac{\partial x}{\partial X_{s}}dX_{s}+\frac{\partial x}{\partial Y_{s}}dY_{s}+\frac{\partial x}{\partial Z_{s}}dZ_{s}+\frac{\partial x}{\partial \varphi }d\varphi +\frac{\partial x}{\partial \omega }d\omega +\frac{\partial x}{\partial \kappa }d\kappa +\left(x\right)-x,\\ v_{y}=\frac{\partial y}{\partial X_{s}}dX_{s}+\frac{\partial y}{\partial Y_{s}}dY_{s}+\frac{\partial y}{\partial Z_{s}}dZ_{s}+\frac{\partial y}{\partial \varphi }d\varphi +\frac{\partial y}{\partial \omega }d\omega +\frac{\partial y}{\partial \kappa }d\kappa +\left(y\right)-y, \end{array}$$

where *v_x_*, *v_y_* are observation corrections, and the above formula can be written in matrix form:

(10) $$\left[\begin{array}{c} v_{x}\\ v_{y} \end{array}\right]=\left[\begin{array}{c} \begin{array}{cc} \begin{array}{ccc} \frac{\partial x}{\partial X_{s}} & \frac{\partial x}{\partial Y_{s}} & \frac{\partial x}{\partial Z_{s}} \end{array} & \begin{array}{ccc} \frac{\partial x}{\partial \varphi } & \frac{\partial x}{\partial \omega } & \frac{\partial x}{\partial \kappa } \end{array} \end{array}\\ \begin{array}{cc} \begin{array}{ccc} \frac{\partial y}{\partial X_{s}} & \frac{\partial y}{\partial Y_{s}} & \frac{\partial y}{\partial Z_{s}} \end{array} & \begin{array}{ccc} \frac{\partial y}{\partial \varphi } & \frac{\partial y}{\partial \omega } & \frac{\partial y}{\partial \kappa } \end{array} \end{array} \end{array}\right]{\left[\begin{array}{cc} \begin{array}{ccc} dX_{s} & dY_{s} & dZ_{s} \end{array} & \begin{array}{ccc} d\varphi & d\omega & d\kappa \end{array} \end{array}\right]}^{T}-\left[\begin{array}{c} x-\left(x\right)\\ y-\left(y\right) \end{array}\right],$$

(11) $$V=\left[\begin{array}{c} v_{x}\\ v_{y} \end{array}\right],$$

(12) $$A=\left[\begin{array}{c} \begin{array}{cc} \begin{array}{ccc} \frac{\partial x}{\partial X_{s}} & \frac{\partial x}{\partial Y_{s}} & \frac{\partial x}{\partial Z_{s}} \end{array} & \begin{array}{ccc} \frac{\partial x}{\partial \varphi } & \frac{\partial x}{\partial \omega } & \frac{\partial x}{\partial \kappa } \end{array} \end{array}\\ \begin{array}{cc} \begin{array}{ccc} \frac{\partial y}{\partial X_{s}} & \frac{\partial y}{\partial Y_{s}} & \frac{\partial y}{\partial Z_{s}} \end{array} & \begin{array}{ccc} \frac{\partial y}{\partial \varphi } & \frac{\partial y}{\partial \omega } & \frac{\partial y}{\partial \kappa } \end{array} \end{array} \end{array}\right],$$

(13) $$X={\left[\begin{array}{cc} \begin{array}{ccc} dX_{s} & dY_{s} & dZ_{s} \end{array} & \begin{array}{ccc} d\varphi & d\omega & d\kappa \end{array} \end{array}\right]}^{T},$$

(14) $$L=\left[\begin{array}{c} x-\left(x\right)\\ y-\left(y\right) \end{array}\right].$$

Based on Equations (10)–(13), Equation (9) can be rewritten as:

(15) V=AX−L.

Finally, according to the least squares principle, the extrinsics *X* of a single image are calculated as:

(16) $$A^{T}PAX=A^{T}PL,$$

(17) $$X={\left(A^{T}A\right)}^{-1}A^{T}L.$$

## 5. Experiments

### 5.1. Experimental Data and Environment

**Figure 4:**
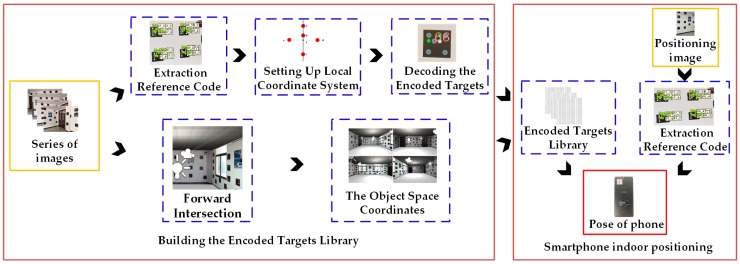
Workflow of smartphone indoor positioning using visual encoded targets.

Figure 4 shows the flowchart of smartphone indoor positioning based on color scatter-encoded target patterns. The solid-line box on the left shows the process of establishing a color scatter-encoded target dataset in the indoor positioning scene, and the solid line box on the right shows the process of smartphone positioning through a single real-time captured image. The yellow box in the left solid-line box shows the sequence images for establishing the color scatter-encoded target dataset. The yellow box in the right solid-line box shows the image captured by the smartphone in the indoor scene, and the red solid-line box shows the calculated pose of the smartphone.

To verify and evaluate the smartphone high-precision indoor positioning dynamic ground truth reference system based on color visual encoded target patterns, a 10 m × 9 m room was selected as an indoor experimental environment, and color encoded target patterns were posted in the room according to certain rules.

**Figure 5:**
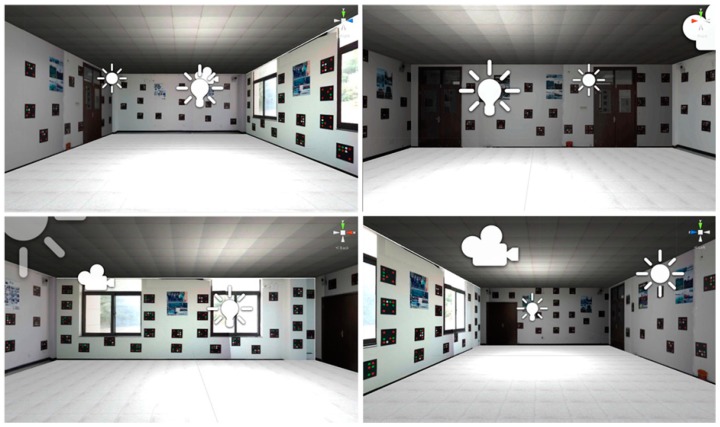
Three-dimensional texture visualization mode indoor scene.

The three-dimensional texture model shown in Figure 5 was made by Unity3D software (Unity Technologies, San Francisco, CA, USA, Version 4.6) according to the indoor experimental environment. The experimental smartphones were a Samsung Galaxy S8 (Huizhou city, China) smartphone with 64 GB storage, eight-core Qualcomm Snapdragon 835 processor, and 2960 × 1440 camera resolution, and Huawei P10 (Dongguan City, China) smartphone with 64 GB storage, eight-core Kirin 960 processor, and 1920 × 1080 camera resolution.

### 5.2. Analysis of Experimental Results

#### 5.2.1. Decoding Color Encoded Target Patterns of Sequence Images and Results of Dataset

**Figure 6:**
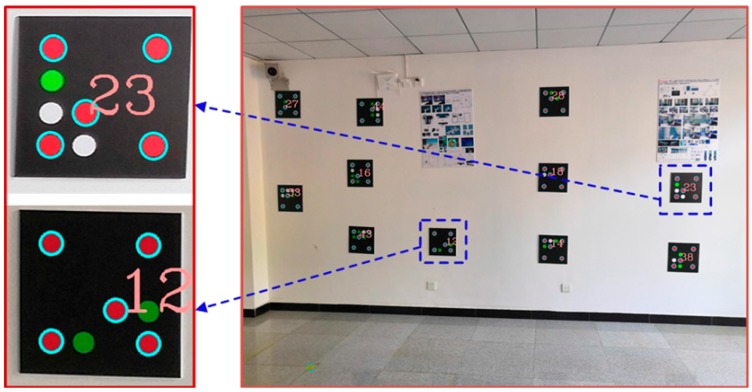
Color-coded mark recognition and decoding.

**Figure 7:**
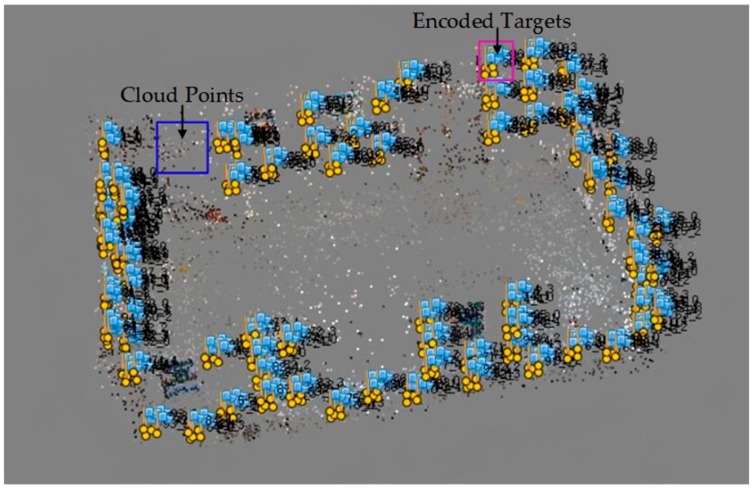
Point cloud 3D display of color encoded targets and feature points.

**Figure 8:**
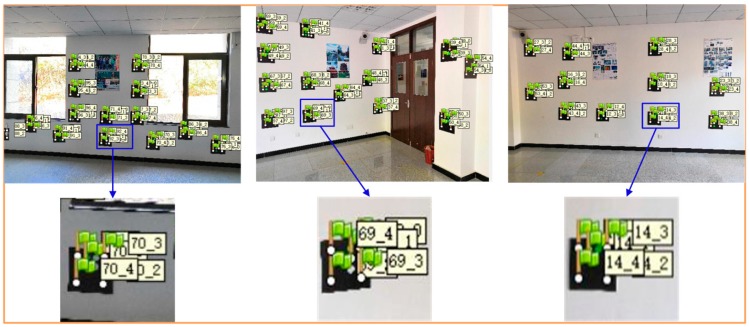
Identified color encoded targets.

**Figure 9:**
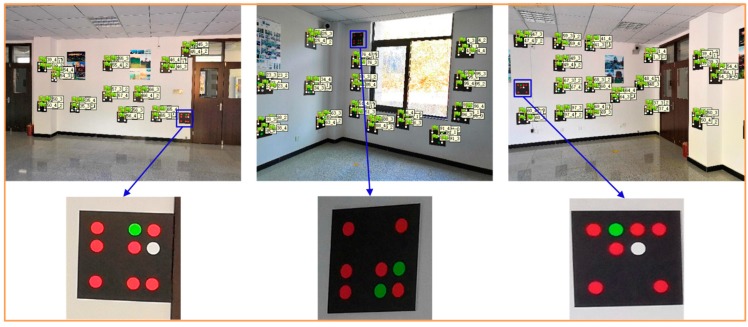
Identified wrong color encoded targets.

Based on certain capturing principles, we used a smartphone to obtain 54 images to establish the color encoded targets dataset, and all of the images were decoded. Figure 6 shows one of the decoded images. All of the recognized red base points are framed by a light blue circle frame, and the decoded values are displayed in a light red font. The results show that the recognition rate of the color scatter encoded targets in 54 images was 100%, indicating that the color encoded targets designed in this paper have a high recognition rate using our recognition algorithm. The color encoded target dataset consisted of 374 encoded values. Figure 7 shows the three-dimensional display of the color scatter encoded targets and the sparse three-dimensional point cloud, where each numbered flag indicates the corresponding color scatter encoded target. The color scatter-encoded targets are clearly consistent with their corresponding point clouds, indicating very high accuracy of color encoded target extraction. In our experiment, the base points extracted in Photoscan were imported into the corresponding images. In Figure 8, flags indicates the imported base points. The results show that the correct rate of extraction of the color encoded target base points is 100%. To verify the fault tolerance of the proposed method, we deliberately added three color scatter-encoded target patterns with base points of the wrong structure. The three wrong patterns were identified and rejected during decoding. Figure 9 shows the experimental results. These demonstrate that the color encoded target patterns designed in this paper are stable in structure, high in fault tolerance, and easily identified and decoded.

#### 5.2.2. Results of Smartphone Positioning

**Figure 10:**
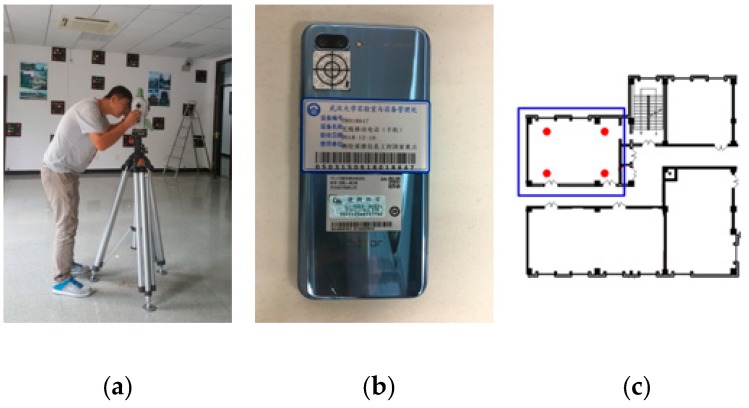
Experimental equipment and environment. (**a**) Leica TS60 measurement robot. (**b**) Ring crosshair on smartphone. (**c**) Control point.

Two smartphones were used to calculate the pose of the single image in the condition of different illumination and angles. A Leica TS60 (Leica, Basel, Switzerland) measurement robot was used to measure the smartphone pose, and its measuring result was used as the ground truth of smartphone positioning. It was difficult to measure the camera on the smartphone because the surface of the camera was a glass material. Therefore, a ring crosshair was affixed to the camera for aiming and automatic tracking measurement of the measurement robot, as shown in Figure 10. Figure 10a shows the Leica TS60 measurement robot, and Figure 10b shows the ring crosshair affixed on the smartphone camera. In Figure 10c the blue box represents the experimental room, and the four red dots are the locations of the four control points in the local indoor coordinate system.

**Figure 11:**
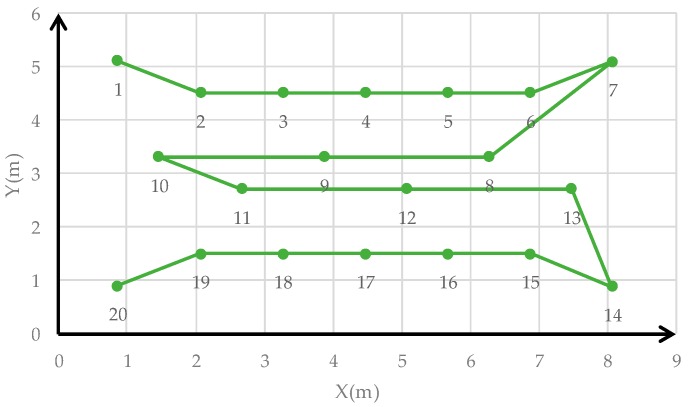
Distribution of 20 positioning points.

**Figure 12:**
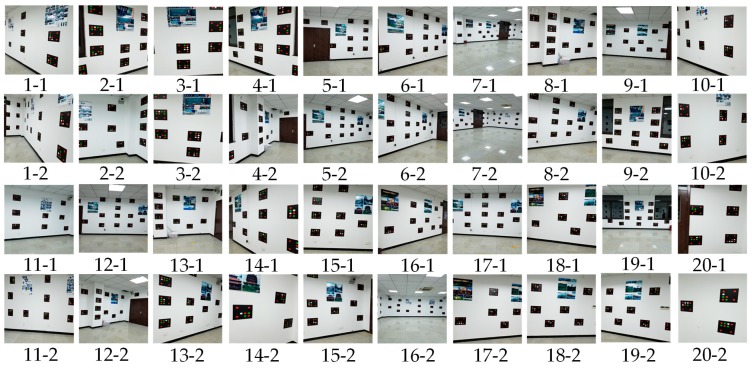
Images of Samsung Galaxy S8 smartphone.

**Figure 13:**
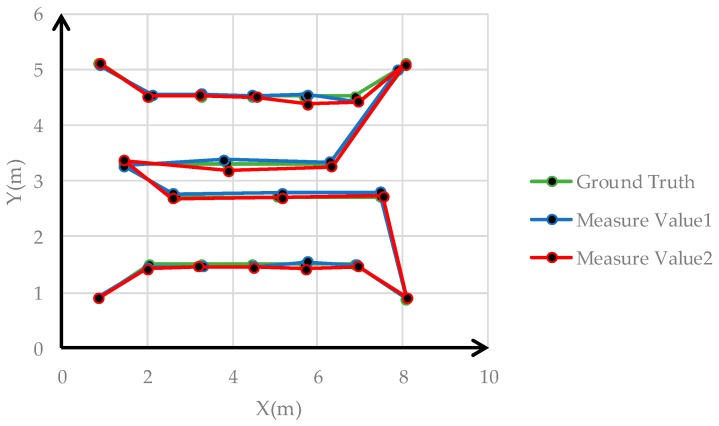
Comparison of two measurements results of the Samsung Galaxy S8 and corresponding ground truth.

**Figure 14:**
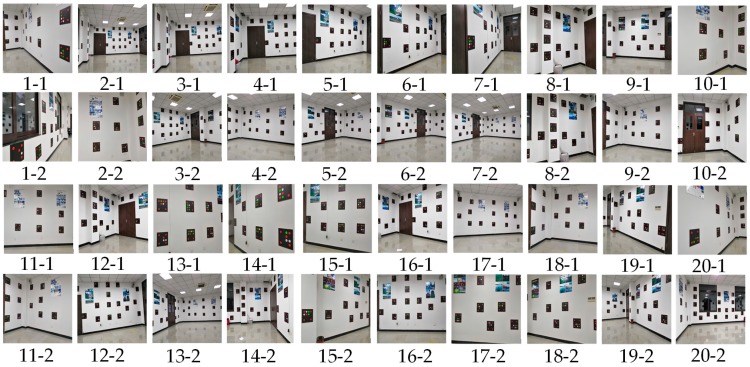
Images of the Huawei P10.

**Figure 15:**
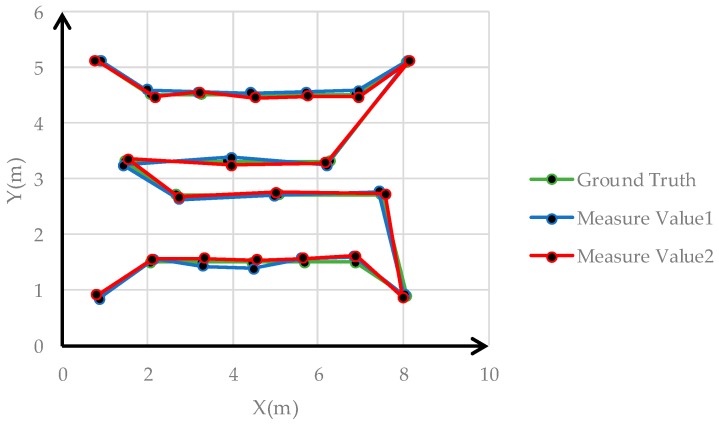
Comparison of measurements results of the Huawei P10 and corresponding ground truth.

Samsung Galaxy S8 smartphones and Huawei smartphones were used to capture images at 20 points to implement the smartphone monolithic positioning experiment, and two images were captured at different orientations at each point. Figure 11 shows the distribution of 20 positioning points. The positioning results calculated by the method based on color visual encoded target patterns were compared with the ground truth measured by the measuring robot. And the Root Mean Square Error (RMSE) values of the *X* direction, *Y* direction, and overall coordinates were calculated. We used Samsung Galaxy S8 and Huawei P10 smartphone to capture images toward an indoor environment wall at the same position, and the positioning results were calculated in real time. Table 1 shows the error among two measurements results and the corresponding ground truth of Samsung Galaxy S8 smartphone. Figure 12 shows images obtained by the Samsung Galaxy S8 smartphone at 20 points at two capturing orientation. Figure 13 shows the comparison of the two measurements results of Samsung Galaxy S8 smartphone and corresponding ground truth at each point. Table 2 shows the error of the two measurements results and the corresponding ground truth of HUAWEI P10 smartphone. Table 3 shows the numbers of points with different accuracy based on the error values of the positioning results of two smartphones. Figure 14 shows images obtained by the HUAWEI P10 at 20 points at two capturing orientations. Figure 15 shows the comparison of the two measurements results of HUAWEI P10 smartphone and corresponding ground truth at each point. Table 4 shows the RMSE values of the Samsung Galaxy S8 smartphone and HUAWEI P10 smartphone in the X direction, Y direction, and overall coordinates.

From the perspective of overall coordinate accuracy, the positioning accuracy of the proposed method is at the centimeter level, which is much better than that of other high-availability indoor positioning technologies. Combined with Table 1, Table 2 and Table 3, the positioning error of 5% of images of the two measurement results of the Samsung Galaxy S8 smartphone in the X direction was greater than 10 cm, the positioning error of 87.5% of images was between 1 cm and 10 cm, and the positioning error of 7.5% of images was less than 1 cm. The positioning error of 5% of images of the two measurement results of the Samsung Galaxy S8 in the Y direction was greater than 10 cm, the positioning error of 77.5% of images was between 1 cm and 10 cm, and the positioning error of 17.5% of images was less than 1 cm. The positioning error of 5% of images of the two measurement results of the Huawei P10 smartphone in the X direction was greater than 10 cm, the positioning error of 92.5% of images is between 1 cm and 10 cm, and the positioning error of 2.5% of images is less than 1 cm. The positioning error of 5% of images of the two measurement results of the Huawei P10 smartphone in the Y direction was greater than 10 cm, the positioning error of 87.5% of images was between 1 cm and 10 cm, and the positioning error of 7.5% of images was less than 1 cm. This shows that the positioning accuracy of color encoded target patterns designed in this paper is high, and the overall positioning accuracy is at the centimeter level. From Table 4, the RMSE values of the two measurement positioning results of the Samsung Galaxy S8 smartphone were roughly the same in the x and y direction, and the RMSE value of the overall coordinates of the two measurements positioning results was around 0.08 m. The RMSE value of the two measurement positioning results of the Huawei P10 smartphone was roughly the same in the x and y direction, and the RMSE value of the overall coordinates of the two measurement positioning results was around 0.09 m. This demonstrates that the accuracy of visual positioning of smartphones in different orientations in the same position is stable, and it also shows that proposed method has good robustness for the pose solution of smartphone positioning images at different orientation. In addition, the RMSE value of the Samsung Galaxy S8 smartphone was slightly lower than that of the Huawei P10 smartphone based on the above data. However, the RMSE values of the positioning results of the two types of smartphones were both less than 0.1 m the different conditions, and the difference was very small. This illustrates that the proposed method has good applicability using two different smartphones, and their positioning accuracy was consistent. In Figure 13, errors of points 5, 7, 9, and 16 were larger than those of other points. In Figure 15, the errors of points 2, 9, 15, and 17 exceeded those of other points. From the corresponding smartphone positioning images in Figure 12 and Figure 14, the capturing distances of these points were far, which indicates that the capturing distance of positioning images has a certain influence on the positioning result. It also conforms to the fundamentals of image positioning technology. However, in a certain distance range, due to the high recognition rate of color encoded patterns in this paper, this adverse effect is weakened. In summary, the proposed method for the smartphone indoor visual positioning based on color encoded target patterns has high positioning accuracy and strong robustness, and the applicability to different smartphones is good.

## 6. Conclusions

There are problems associated with directly dynamically measuring smartphone poses using current indoor positioning ground truth reference systems and high-price deployments. To address these problems, this paper proposes a kind of high recognition rate, large encoding capacity and good robustness color visual scatter-encoded patterns as a smartphone indoor positioning ground truth reference system for meeting the needs of frequently, freely, and simply obtaining the accuracy of indoor positioning tests of smartphones with a low cost in daily experiments. While using other positioning methods to carry out a smartphone positioning experiment at the same time, the proposed true value reference system could dynamically self-locate the instantaneous pose of a smartphone in real-time. Compared with existing artificial encoded targets, the structure of the color encoded targets designed in this paper is the key to ensure the high-precision positioning result of a single image of a smartphone. The structure of the base points ensures that the color encoded target patterns maintain stability of rotation, translation, and zooming during affine transformation. The color encoded target patterns of this paper combine geometric structure and color information to increase the encoding capacity; to meet the positioning experimental needs of most indoor scenes. Experiments show that the color encoded target patterns effectively simplify the decoding of encoded targets, and the recognition rate of the proposed encoded targets is 100%. Furthermore, the proposed algorithm is robust. Experiments show that its positioning results have good applicability to different smartphone cameras and capturing angles, and the smartphone positioning requirements for lighting conditions are also relatively loose. In our experiment, the positioning accuracy of the system could reach the centimeter level, which is far better than current high-availability and low-cost Bluetooth, Wi-Fi and other positioning sources. This reference system is low-cost and has good real-time dynamics. Therefore, we recommend it as the ground truth reference system for other smartphone indoor positioning technologies.

## Figures and Tables

**Table 1:** Positioning accuracy of the Samsung Galaxy S8 smartphone.

Image No	Ground Truth		Measure Value1				Measure Value2			
	*X* (m)	*Y* (m)	*X* (m)	*Y* (m)	*Δx* (m)	*Δy* (m)	*X* (m)	*Y* (m)	*Δx* (m)	*Δy* (m)
1	0.864	5.112	0.8907	5.0693	−0.0267	0.0427	0.9179	5.1008	0.0539	0.0112
2	2.066	4.511	2.1173	4.5479	−0.0513	−0.0369	2.0270	4.5205	−0.0390	−0.0095
3	3.268	4.511	3.2864	4.5569	−0.0184	−0.0459	3.2485	4.5315	0.0195	−0.0205
4	4.47	4.511	4.4676	4.5325	0.0024	−0.0215	4.5607	4.5037	−0.0907	0.0073
5	5.672	4.511	5.7526	4.5447	−0.0806	−0.0337	5.7527	4.3770	−0.0807	0.1340
6	6.874	4.511	6.9553	4.4239	−0.0813	0.0871	6.9493	4.4220	−0.0753	0.0890
7	8.078	5.102	7.8883	5.0043	0.1897	0.0977	8.0601	5.0807	0.0179	0.0213
8	6.273	3.309	6.2950	3.3367	−0.0220	−0.0277	6.3198	3.2584	−0.0468	0.0506
9	3.869	3.309	3.8038	3.3845	0.0652	−0.0755	3.8976	3.1815	−0.0286	0.1275
10	1.465	3.309	1.4411	3.2747	0.0239	0.0343	1.4664	3.3622	−0.0014	−0.0532
11	2.667	2.708	2.6206	2.7658	0.0464	−0.0578	2.5917	2.6807	0.0753	0.0273
12	5.071	2.708	5.1615	2.7827	−0.0905	−0.0747	5.1848	2.6993	−0.1138	0.0087
13	7.477	2.708	7.4664	2.8056	0.0106	−0.0976	7.5248	2.7351	−0.0478	−0.0271
14	8.071	0.886	8.0822	0.8911	−0.0112	−0.0051	8.0935	0.9128	−0.0225	−0.0268
15	6.874	1.506	6.9288	1.4962	−0.0548	0.0098	6.9537	1.4712	−0.0797	0.0348
16	5.672	1.506	5.7577	1.5511	−0.0439	0.0421	5.7068	1.4193	−0.0348	0.0867
17	4.47	1.506	4.5152	1.4590	−0.0857	−0.0451	4.5133	1.4521	−0.0433	0.0539
18	3.268	1.506	3.3119	1.4639	−0.0439	0.0421	3.1836	1.4661	0.0844	0.0399
19	2.066	1.506	2.0288	1.4544	0.0372	0.0516	2.0082	1.4213	0.0578	0.0847
20	0.861	0.905	0.8475	0.9129	0.0135	−0.0079	0.8526	0.9008	0.0084	0.0042

**Table 2:** Positioning accuracy of the Huawei P10 smartphone.

Image No	Ground Truth		Measure Value1				Measure Value2			
	*X* (m)	*Y* (m)	*X* (m)	*Y* (m)	*Δx* (m)	*Δy* (m)	*X* (m)	*Y* (m)	*Δx* (m)	*Δy* (m)
1	0.864	5.112	0.9244	5.1105	−0.0604	0.0015	0.7704	5.1305	0.0936	−0.0185
2	2.066	4.511	1.9848	4.6053	0.0812	−0.0943	2.1661	4.4673	−0.1001	0.0437
3	3.268	4.511	3.1882	4.5621	0.0798	−0.0511	3.2332	4.5516	0.0348	−0.0406
4	4.47	4.511	4.4122	4.5488	0.0578	−0.0378	4.5294	4.4546	−0.0594	0.0564
5	5.672	4.511	5.7249	4.5530	−0.0529	−0.0420	5.7495	4.4855	−0.0775	0.0255
6	6.874	4.511	6.9493	4.5870	−0.0753	−0.0760	6.9697	4.4711	−0.0957	0.0399
7	8.078	5.102	8.1159	5.1317	−0.0379	−0.0297	8.1415	5.1075	−0.0635	−0.0055
8	6.273	3.309	6.1945	3.2366	0.0785	0.0724	6.1769	3.2822	0.0961	0.0268
9	3.869	3.309	3.9530	3.3876	−0.0840	−0.0786	3.9573	3.2393	−0.0883	0.0697
10	1.465	3.309	1.4264	3.2418	0.0386	0.0672	1.5478	3.3625	−0.0828	−0.0535
11	2.667	2.708	2.7542	2.6276	−0.0872	0.0804	2.7397	2.6715	−0.0727	0.0365
12	5.071	2.708	4.9918	2.6933	0.0792	0.0147	5.0151	2.7578	0.0559	−0.0498
13	7.477	2.708	7.4272	2.7742	0.0498	−0.0662	7.5758	2.7342	−0.0988	−0.0262
14	8.071	0.886	8.0187	0.9193	0.0523	−0.0333	7.9943	0.8785	0.0767	0.0075
15	6.874	1.506	6.8491	1.5988	0.0249	−0.0928	6.8849	1.6132	−0.0109	−0.1072
16	5.672	1.506	5.6116	1.5780	0.0604	−0.0720	5.6295	1.5732	0.0425	−0.0672
17	4.47	1.506	4.4905	1.3888	−0.0205	0.1172	4.5813	1.5490	−0.1113	−0.0430
18	3.268	1.506	3.3080	1.4268	−0.0400	0.0792	3.3397	1.5708	−0.0717	−0.0648
19	2.066	1.506	2.1617	1.5620	−0.0957	−0.0560	2.1078	1.5623	−0.0418	−0.0563
20	0.861	0.905	0.8643	0.8492	−0.0033	0.0558	0.8099	0.9172	0.0511	−0.0122

**Table 3:** The number of different accuracy positioning points of the Samsung Galaxy S8 smartphone and Huawei P10 smartphone.

Phone Type		Samsung Galaxy S8				Huawei P10			
		*Δx* (m)		*Δy* (m)		*Δx* (m)		*Δy* (m)	
		Measure1 (Number)	Measure2 (Number)	Measure1 (Number)	Measure2 (Number)	Measure1 (Number)	Measure2 (Number)	Measure1 (Number)	Measure2 (Number)
Error (cm)	>10	1	1	0	2	0	2	1	1
	1–10	18	17	17	14	19	18	18	17
	<1	1	2	3	4	1	0	1	2

**Table 4:** Root mean square error (RMSE) of positioning results.

Phone Type		Samsung Galaxy S8		Huawei P10	
		Measure Value1	Measure Value2	Measure Value1	Measure Value2
RMSE	*Δx* (m)	0.0650	0.0591	0.0629	0.0757
	*Δy* (m)	0.0543	0.0597	0.0669	0.0488
	*Δd* (m)	0.0846	0.0840	0.0918	0.0900
